# Usefulness of Adaptive Complex Signal Average Diffusion-weighted MR Imaging of the Abdomen

**DOI:** 10.2463/mrms.mp.2026-0009

**Published:** 2026-07-25

**Authors:** Miwa Matsukuma, Masahiro Tanabe, Mayumi Higashi, Masatoshi Yamane, Masahiro Abe, Ryutaro Yano, Haruki Furutani, Jo Ishii, Katsuyoshi Ito

**Affiliations:** 1 Department of Radiology, Yamaguchi University Graduate School of Medicine, Ube, Yamaguchi, Japan; 2 Department of Radiological Technology, Yamaguchi University Hospital, Ube, Yamaguchi, Japan; 3 Canon Medical Systems Corporation, Otawara, Tochigi, Japan

**Keywords:** apparent diffusion coefficient, diffusion-weighted imaging, numbers of excitations, signal intensity, signal-to-noise ratio

## Abstract

**Purpose:**

To evaluate the clinical feasibility and quantitative performance of adaptive complex signal average (ACSA) diffusion-weighted imaging (DWI) in abdominal MRI and to compare ACSA DWI with conventional non-ACSA DWI in terms of signal intensity (SI), SNR, apparent diffusion coefficient (ADC), signal intensity difference ratio (SIDR), and lesion contrast across different numbers of excitations (NEX).

**Methods:**

This retrospective study included 82 patients who underwent free-breathing abdominal DWI on a 3T system between January and July 2025. ACSA DWI was reconstructed from the same raw data as non-ACSA DWI using a combination of adaptive averaging and complex-domain averaging. ROIs were placed in the liver, pancreas, left adrenal gland, and spinal muscles to measure SI, SNR, and ADC. SIDR and inter-lobar right–left (R–L) ratio in SI and ADC were calculated at NEX 2, 3, and 4. For 37 patients with non-cystic hepatic nodules, lesion SI, ADC, contrast ratio, and contrast-to-noise ratio (CNR) were assessed.

**Results:**

ACSA DWI significantly increased SI and SNR in motion-prone organs, including the lateral hepatic segment, pancreas, and left adrenal gland, at all NEX levels (*P* < 0.05). ADC values in abdominal parenchymal organs and hepatic nodules were lower with ACSA DWI, reflecting the noise-floor suppression and adaptive average effects. R–L SI and ADC ratios were significantly different and were closer to 1 in ACSA DWI, indicating improved hepatic signal homogeneity. Lesion SI, contrast ratio, and CNR were significantly higher with ACSA DWI, enhancing nodule conspicuity.

**Conclusion:**

ACSA DWI improves signal uniformity, SNR, and lesion contrast while maintaining reasonable ADC quantification. Without prolonging scan time or modifying acquisition parameters, ACSA provides a robust, clinically feasible post-processing technique for enhancing both image quality and quantitative reliability in abdominal DWI.

## Introduction

Diffusion-weighted imaging (DWI) of the abdomen plays an essential role in evaluating parenchymal organs and detecting malignant lesions by characterizing tissue diffusivity.^1^^–^^4^ However, in conventional DWI, because the signals from each excitation are equally weighted and added, signal reduction can occur in regions where the signal differs for each excitation due to the effects of cardiac pulsation and respiratory motion, such as the left lateral segment of the liver.^5^^–^^8^ Such motion-related artifacts result in poor signal uniformity, increased image noise and inaccurate apparent diffusion coefficient (ADC) values, limiting the diagnostic reliability of abdominal DWI.

Various strategies have been developed to address these problems, including navigator-based motion correction and multi-shot echo-planar imaging (EPI).^6^^,^^9^^–^^11^ Although these approaches can mitigate motion artifacts, they often increase scan time, require additional sequence optimization, or risk altering quantitative diffusion parameters. Therefore, a post-processing method that can reduce noise and motion-related signal degradation without modifying the acquisition protocol would be highly valuable in clinical practice.

Recently, 2 novel reconstruction techniques have been introduced. First, adaptive averaging applies a weighted average based on the signal amplitude of each excitation, thereby reducing the influence of low-intensity signals caused by cardiac and respiratory motion and enhancing the robustness of the resulting averaged signal.^12^^–^^14^ Second, complex signal average operates in the complex domain to suppress the noise floor caused by the Rician distribution inherent to magnitude DWI.^15^^–^^17^ This dual approach allows selective enhancement of true signal components, potentially leading to improved image quality and diagnostic confidence without increasing acquisition time. However, to our knowledge, there have been no clinical studies investigating the combined use of these 2 image reconstruction methods. Because a prototype adaptive complex signal average (ACSA) reconstruction method combining 2 image reconstruction techniques became available at Yamaguchi University Hospital, this study evaluated the clinical feasibility and quantitative performance of ACSA DWI in abdominal imaging, and compared it with conventional non-ACSA DWI in terms of signal intensity (SI), SNR, ADC, signal intensity difference ratio (SIDR), and lesion contrast. We also examined the influence of different numbers of excitations (NEX) on image performance to clarify the optimal settings for ACSA reconstruction.

## Materials and Methods

Two authors (M.A. and R.Y.) are employees of Canon Medical Systems and provided technical information relevant to the study. They had no role in the study design, data collection, data analysis, or interpretation of the data.

### Study population

This retrospective, single-center study was conducted between January 2025 and July 2025. Institutional Review Board of Yamaguchi University Hospital approved this study and waived the requirement for informed consent due to the retrospective nature of the analysis. Inclusion criteria into this study were as follows: ≥ 18 years of age; imaging performed on one of our four 3T MRI scanners—specifically, the Canon system (Vantage Centurian; Canon Medical Systems, Tochigi, Japan), which is capable of ACSA reconstruction (abdominal MR examinations were randomly assigned to one of the four 3T MRI systems); and patients undergoing free-breathing DWI of the abdomen (adaptive complex signal average reconstruction was applied as post-processing for all patients). Exclusion criteria from this study were as follows: patients with a history of surgery in the left lobe of the liver or pancreas (n = 4); patients with pancreatic cancer and impaired assessment of the normal pancreas (n = 5); patients with severe hepatic hemosiderosis where signal measurement is difficult due to marked signal drop (n = 2); patients with diffuse metastatic liver infiltration or large hepatic tumors that could interfere with signal measurement (n = 2); patients with poor image quality (n = 1); and duplicate cases (n = 7). Consequently, 82 patients were included in the final analysis (53 males, 29 females, mean age 69, range 31–91) (Fig. 1). Among these, 37 patients had non-cystic hepatic nodules including hepatocellular carcinoma (HCC) (n = 20), metastasis from cancers of colon, rectum or pancreas (n = 4), hemangioma (n = 12), and focal nodular hyperplasia (FNH) (n= 1). The mean size of these lesions was 20.6 ± 13.5 mm (range: 6–76 mm). These lesions were diagnosed histologically (n = 8) or clinically based on a combination of available imaging modalities, including CT and other MR sequences with characteristic imaging features (n = 29).

### MRI technique

All MR examinations were performed on a clinical 3T MR system (Vantage Centurian) with a 16-channel body phased-array coil. Free-breathing DWI using 2D, spin-echo (SE) EPI sequence was performed as a part of abdominal MR protocol. The imaging parameters were as follows: TR/ TE, 1663/51 msec; flip angle, 90°; FOV, 280 × 380 mm; acquisition matrix, 200 × 128; reconstruction matrix, 600 × 816 by using Precise IQ Engine (PIQE) algorithm^18^; bandwidth, 3125 Hz/pixel; slice thickness, 5 mm; number of slices, 35; b-values, 0 s/mm^2^ and 1000 s/mm^2^; parallel imaging factor, 2; number of excitations (NEX) = 2 (n = 21), 3 (n = 29), or 4 (n = 32); acquisition time, 85, 120, or 135 sec. Each NEX group (NEX = 2, 3, 4) was assigned based on a date-based basis to optimize image quality in a clinical setting. After conventional DWI (non-ACSA DWI) images reconstructed with magnitude averaging were obtained, ACSA DWI images were reconstructed with a post-processing technique using same DWI data. In this study, the receiver gain, etc. were the same for each image reconstruction.

### Image analysis

Quantitative image analysis for 2 sets of DWI images was performed in random order using a picture archiving and communication system (PACS; Shade Quest; FUJIFILM Medical Solutions, Tokyo, Japan) by a radiologist (M.M.) with 18 years of experience and checked by a radiologist (K.I.) with 37 years of experience in abdominal imaging. All were blinded to the MRI parameters, clinical information such as patient history, final diagnoses, and findings of other imaging modalities.

Circular ROIs were drawn manually on the liver (periphery of left lateral segment, center of left lateral segment, periphery of right lobe, and center of right lobe), pancreas (head, body, and tail), left adrenal gland, and erector spinae muscles. The right adrenal gland is a very thin and narrow organ, making precise ROI measurement difficult; therefore, ROI measurement was not performed. ROIs were placed at identical locations on the DWI image and ADC map of both ACSA and non-ACSA DWI using copy and paste functions.

ROIs measurement was used to determine SI, SNR (SI of organ/standard deviation [SD] of muscle) and ADC values in each organ and compared between ACSA and non-ACSA DWI. SIDR ([SI of ACSA − SI of non-ACSA]/SI of non-ACSA × 100) was also analyzed according to differences in the number of excitations (NEX 2, 3, and 4). Additionally, differences in SI and ADC values between the right lobe and left lateral segment for ACSA DWI and non-ACSA DWI at NEX of 2, 3, or 4 were calculated according to the following formulas: right–left (R–L) SI ratio = (SI of liver, right lobe, center)/(SI of liver, lateral segment, periphery), and R–L ADC ratio = (ADC of liver, right lobe, center)/(ADC of liver, lateral segment, periphery).

Regarding non-cystic hepatic nodules, one largest lesion per lesion type was analyzed. ROI was placed centrally in each lesion on a single representative slice (reference slice), which was largely unaffected by motion and susceptibility artifacts and pixel misalignments. For lesions with central necrosis or cystic components, the ROI placements were performed with exclusion of such areas. Then, SI, ADC values, contrast ratio (CR) (SI of nodule − SI of liver), and contrast-to-noise ratio (CNR) ([SI of nodule − SI of liver]/SD of muscle) were measured and calculated.

### Statistical analysis

Continuous variables are presented as mean ± SD or median (interquartile range). Normality was tested with the Shapiro–Wilk test. Age and body weight were compared among the NEX 2, 3, and 4 groups using the Kruskal–Wallis test, and the distribution of disease categories was evaluated using Fisher’s exact test. The Wilcoxon signed-rank test was used to compare the values of SI, SNR, ADC, CR, CNR, R–L SI ratio and R–L ADC ratio between ACSA DWI and non-ACSA DWI images. For SIDR NEX 2, 3, and 4, the Kruskal–Wallis test was used for comparisons with Bonferroni-corrected Steel–Dwass test for post-hoc analysis. All statistical analyses were performed using JMP version 19 (JMP Statistical Discovery LLC, Cary, NC, USA), and *P*-values < 0.05 were considered statistically significant.

## Results

Of the 82 patients, DWI was acquired with NEX = 2 in 21 patients, NEX = 3 in 29 patients, and NEX = 4 in 32 patients. No significant differences in age (*P* = 0.83) or body weight (*P* = 0.49) were observed among the NEX groups. In each NEX group, 9 of 21 patients in NEX 2, 9 of 29 in NEX 3, and 9 of 32 in NEX 4 had chronic liver disease or cirrhosis. Each NEX group included hepatic, biliary, and pancreatic lesions. Hepatic lesions included HCCs, metastatic liver tumors, hemangiomas, hepatic cysts, and FNH, and were distributed as 13/14/19 across the NEX 2/3/4 groups. Biliary lesions included gallbladder cancer, cholelithiasis, choledocholithiasis, and primary sclerosing cholangitis, and were distributed as 3/5/3. Pancreatic lesions included small pancreatic cancer, pancreatic cystic lesions, and focal autoimmune pancreatitis, and were distributed as 5/10/10. No significant differences were observed in the distribution of these disease groups (*P* = 0.80).

In ACSA DWI, the SI of the lateral hepatic segment (both peripheral and central regions), peripheral right hepatic lobe, pancreas, and left adrenal gland were significantly higher than those of non-ACSA DWI at all NEX levels (2, 3, and 4) (*P* < 0.05) (Figs. 2 and 3). The improvement in SI was particularly evident at NEX 4, reflecting signal stabilization with increasing excitations. In contrast, the erector spinae muscles showed no significant difference in SI between the 2 techniques. The central region of the right hepatic lobe also showed no significant difference at NEX 2 and 3 (Table 1).

In the comparison of SNR of each organ, the SNR was significantly higher in ACSA DWI for the lateral hepatic segment, pancreas, and left adrenal gland compared with non-ACSA DWI at all NEX levels (2, 3, and 4) (*P* < 0.05) (Table 2). However, the central right hepatic lobe exhibited no significant SNR differences at all NEX. The erector spinae muscles also exhibited no significant differences in SNR at NEX 2 and 3, except for NEX 4. These results indicate that adaptive complex signal average effectively enhances SNR in flat, non-voluminous organs influenced by cardiac and respiratory motion and partial volume effects, while volumetric structures tended to show minimal change.

The results of the comparison of ADC values between ACSA DWI and non-ACSA DWI are shown in Table 3. The ADC values of the lateral hepatic segment, right hepatic lobe, pancreas, and left adrenal gland were significantly lower in ACSA DWI than in non-ACSA DWI (*P* < 0.05). Conversely, no significant difference in ADC was observed in the erector spinae muscles. These findings suggest that adaptive complex signal average may slightly reduce ADC values in abdominal parenchymal organs.

The SIDR showed significant differences among NEX 2, 3, and 4 for the lateral hepatic segment (*P* < 0.05) (Table 4). SIDR increased progressively with higher NEX values and was greater at the hepatic periphery than at the center. Significant inter-group differences were also observed in the pancreatic tail and left adrenal gland, whereas the right hepatic lobe, pancreatic head-body, and erector spinae muscles showed no significant SIDR variation across NEX levels.

Table 5 shows the results of the inter-lobar signal and ADC homogeneity of the liver assessed by R–L SI and ADC ratio. Across all NEX levels, the R–L SI ratio was significantly different and was closer to 1 in ACSA DWI than in non-ACSA DWI (all *P* < 0.0001). Similarly, the R–L ADC ratio was significantly different and shifted closer to 1 with ACSA (0.82–0.89) compared with non-ACSA DWI (0.64–0.77), indicating reduction of left-lobe ADC overestimation. Since the liver parenchyma is generally considered to be a homogeneous parenchymal organ, R–L SI and ADC ratios closer to 1 indicate that ACSA DWI substantially corrected the imbalance between the right lobe and left lateral segment by restoring SI and ADC values in the motion-affected left hepatic lobe, thereby producing a more homogeneous parenchymal appearance.

Regarding inter-lobar NEX-dependence of the liver within ACSA (Table 6), the R–L SI ratio showed a significant dependence on the NEX (*P* = 0.0035). The ratio progressively approached 1 as NEX increased (1.56 at NEX 2, 1.29 at NEX 3, and 1.26 at NEX 4), suggesting that repeated excitations further enhanced restoration of left-lobe signal uniformity. The R–L ADC ratio also demonstrated a significant overall trend toward 1 (*P* = 0.0438), increasing from 0.82 at NEX 2 to 0.89 at NEX 3 and 4. Although pairwise comparisons did not reach statistical significance, the consistent shift toward 1 indicates gradual improvement in diffusion uniformity with increasing NEX.

For quantitative analysis of non-cystic hepatic nodules (Table 7), the SI, CR, and CNR were significantly higher in ACSA DWI than in non-ACSA DWI (*P* < 0.05) (Figs. 4 and 5). In contrast, the ADC values of the nodules were significantly lower in ACSA DWI.

## Discussion

The present study demonstrated that ACSA DWI significantly improved abdominal image quality compared with conventional non-ACSA DWI, particularly in motion-prone and smaller organs as well as non-cystic hepatic nodules. The core mechanism of ACSA, which involves adaptive average and complex signal average, appears to effectively mitigate the impact of motion-induced signal dropout^12^^–^^14^ and the Rician noise floor,^15^^,^^16^ thereby improving quantitative diagnostic reliability.

Adaptive average mitigates signal variability caused by cardiac pulsation and differences in acquisition timing, which can lead to signal underestimation relative to the expected value in affected regions. Instead of using a simple arithmetic average across multiple acquisitions, adaptive average uses a weighted averaging strategy based on the SI of each repetition.^12^^–^^14^ The weights are computed from a smoothed image of the original SIs to reflect global signal variations rather than local fluctuations, thereby reducing the impact of low-intensity signals and enhancing the robustness of the resulting averaged signal.

Complex signal average uses phase correction to enable averaging in the complex domain, thereby suppressing the noise floor. In conventional DWI, magnitude reconstruction introduces a positively biased Rician noise distribution, limiting noise reduction even after averaging. By performing phase-corrected real-data reconstruction,^15^^,^^16^ implemented with deep learning, the complex signal average process yields a real-valued image that preserves zero-mean Gaussian noise behavior. As a result, complex signal average prevents elevation of the noise floor and enables more effective noise suppression as NEX increases.

The significant increase in SI and SNR observed in organs susceptible to motion, such as the lateral hepatic segment, pancreas, and left adrenal gland, compared to conventional DWI, strongly supports the efficacy of the adaptive average component of ACSA. These regions often suffer from signal reduction due to cardiac pulsation and respiratory motion,^19^^,^^20^ which the adaptive average successfully suppresses by down-weighting low-intensity, motion-corrupted signals. The greater improvement at higher NEX, as evidenced by the progressive increase in SIDR for the lateral hepatic segment, suggests that a larger pool of excitations allows the adaptive algorithm to more reliably select and average high-quality, high-signal components.

Conversely, organs with minimal physiological motion, like the erector spinae muscles, showed no significant SI or SNR difference. Similarly, the central right hepatic lobe, a volumetric structure generally less susceptible to motion artifacts than the periphery,^12^ showed minimal change, particularly at lower NEX. This regional heterogeneity confirms that ACSA’s primary benefit is in motion-susceptible and flat organs, where it stabilizes signal uniformity.

A crucial finding is the significantly lower ADC values in ACSA DWI for abdominal parenchymal organs compared to non-ACSA DWI. ACSA-DWI theoretically elevates the base signal level of the entire image, leading to the reduction of ADC values compared with non-ACSA DWI. Additionally, by suppressing the Rician noise floor, ACSA DWI removes noise-related signal elevation and yields ADC values that are closer to the inherent diffusion characteristics.^21^^–^^24^

Regarding the inter-lobar analysis of the liver, both the R–L SI ratio and the R–L ADC ratio were significantly closer to 1 with ACSA DWI than with conventional non-ACSA DWI, indicating that ACSA DWI contributes to more homogeneous signal representation within the liver. Because the liver is physiologically a uniform organ, smaller inter-lobar disparities suggest improved accuracy of regional diffusion measurements. Moreover, the increasing NEX further reduced the SI gap between lobes in ACSA DWI, suggesting that ACSA DWI benefits from having multiple excitations, enabling more reliable identification of motion-corrupted repetitions. These results collectively indicate that ACSA DWI enhances within-organ uniformity, a key property when applying diffusion metrics to assess parenchymal diseases.^25^

Finally, the improved SI, CR, and CNR for hepatic nodules underscore the clinical value of ACSA, directly translating to enhanced lesion conspicuity and diagnostic confidence. These improvements, combined with the enhancement of inter-lobar homogeneity, indicate that ACSA DWI provides both global (organ-level) and focal (lesion-level) image quality advantages.

The findings have profound clinical significance. By improving SI uniformity and increasing SNR in motion-prone organs like the liver’s left lobe, pancreas, and adrenal glands, ACSA DWI can reduce the incidence of false-negative diagnoses due to poor image quality and signal dropout.^1^ The enhanced lesion conspicuity for hepatic nodules means better detection and characterization of focal lesions.^2^ Most importantly, ACSA DWI is a post-processing technique that achieves these improvements without increasing the acquisition time or altering the sequence protocol, making it immediately implementable and highly practical in a busy clinical setting.^15^^,^^23^ The production of more stable and potentially more accurate ADC values could lead to better quantitative assessment of tissue properties and treatment response monitoring.^21^^,^^26^

This study has several limitations. First, it was retrospective and single-center in design, including patients with heterogeneous disease backgrounds and variable motion compliance. Second, only 3 NEX levels (2, 3, and 4) were analyzed; the performance of adaptive reconstruction under different b-values and field strengths warrants further validation. Third, the ADC decrease on ACSA DWI is thought to be a reasonable ADC value due to the averaging weighting effect and noise floor correction, but further investigation is needed using phantom experiments and multicenter reproducibility studies. Fourth, while the study demonstrated a positive effect of increasing NEX, the trade-off between the benefit gained from higher NEX and the potential increase in motion sensitivity for extended scan times was not fully explored. Finally, this study did not involve subjective evaluation of image quality (e.g., a 5-point rating by several radiologists) and was unable to evaluate the clinical practicality of ACSA DWI, such as the degree of improvement in the visual signals of organs and lesions. Additionally, lesion evaluation was limited to non-cystic hepatic nodules, and its applicability to pancreatic, renal, or adrenal lesions should be addressed in future research.

## Conclusion

In conclusion, ACSA DWI significantly improves abdominal image quality by enhancing SI, SNR, and lesion contrast, while maintaining reasonable ADC quantification. The technique effectively compensates for motion-related signal loss and reduces the noise floor inherent to DWI, without increasing acquisition time. These findings establish adaptive complex signal average as a robust and clinically feasible post-processing method for improving diagnostic confidence and quantitative integrity in upper abdominal DWI.

## Figures and Tables

**Fig. 1:**
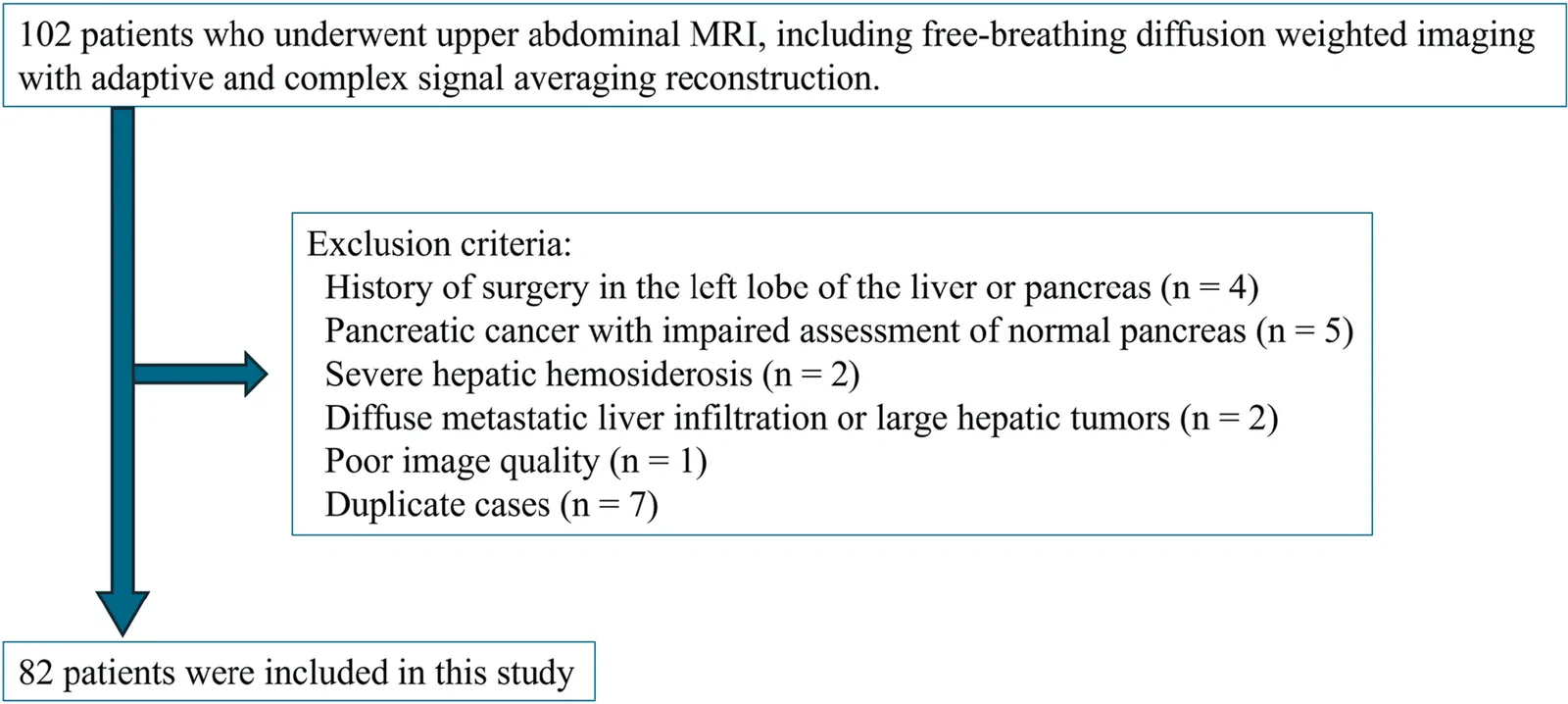
Patient selection flowchart.

**Fig. 2:**
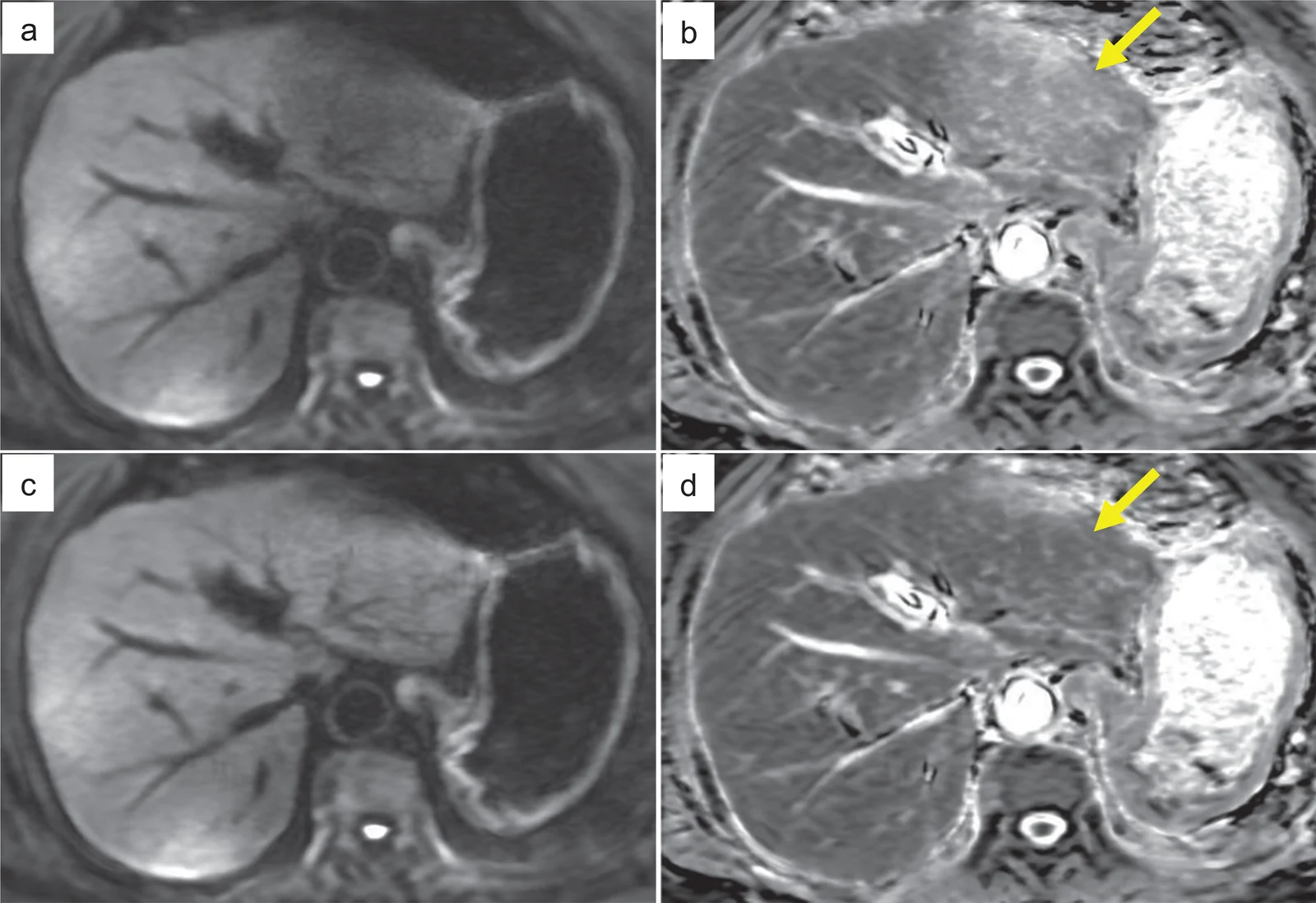
Comparison of (**a**) non-ACSA (conventional) DWI and (**b**) corresponding ADC map and (**c**) ACSA DWI and (**d**) corresponding ADC map (NEX = 4) in an 86-year-old woman. On ACSA DWI, the signal intensity of the liver parenchyma in the left lateral segment showed a distinct signal increase (arrow in **c**) compared to non-ACSA DWI, with signal intensity comparable to that of the right lobe. In the ACSA ADC map, the overestimation of ADC values observed in the lateral segment on the non-ACSA ADC map is improved, and the inter-lobar difference in ADC values is scarcely observed (arrow in **d**). ACSA, adaptive complex signal average; ADC, apparent diffusion coefficient; DWI, diffusion-weighted imaging; NEX, numbers of excitations.

**Fig. 3:**
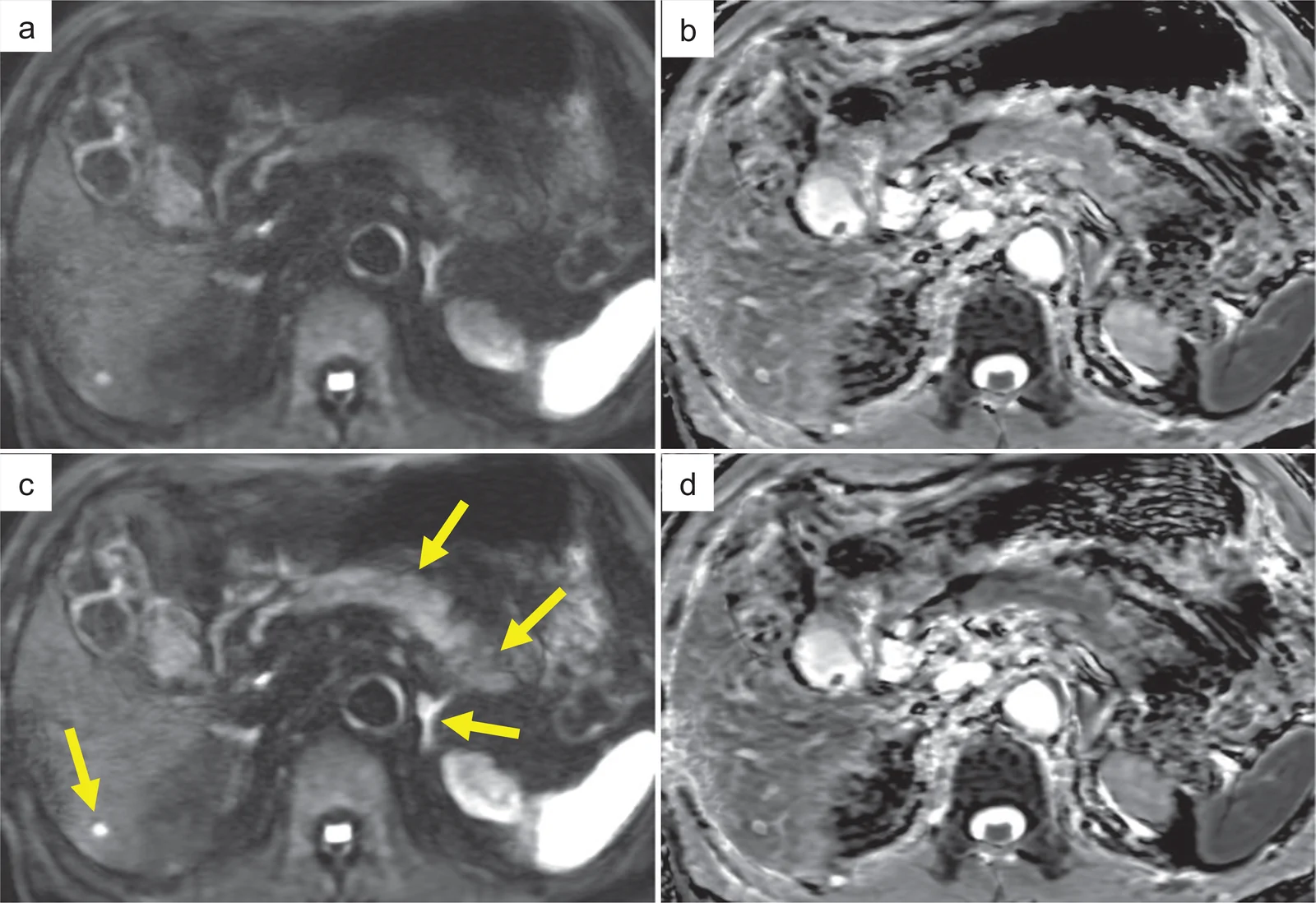
Comparison of (**a**) non-ACSA (conventional) DWI and (**b**) corresponding ADC map and (**c**) ACSA DWI and (**d**) corresponding ADC map (NEX = 4) in a 75-year-old man. Compared with non-ACSA DWI, ACSA DWI showed increased signals in the pancreatic body and tail, left adrenal gland, and hepatic hemangioma in the liver, making these anatomical structures and lesions more distinct (arrows in **c**), but little signal change was observed in the right hepatic lobe and erector spinae muscle. ACSA, adaptive complex signal average; ADC, apparent diffusion coefficient; DWI, diffusion-weighted imaging; NEX, numbers of excitations.

**Fig. 4:**
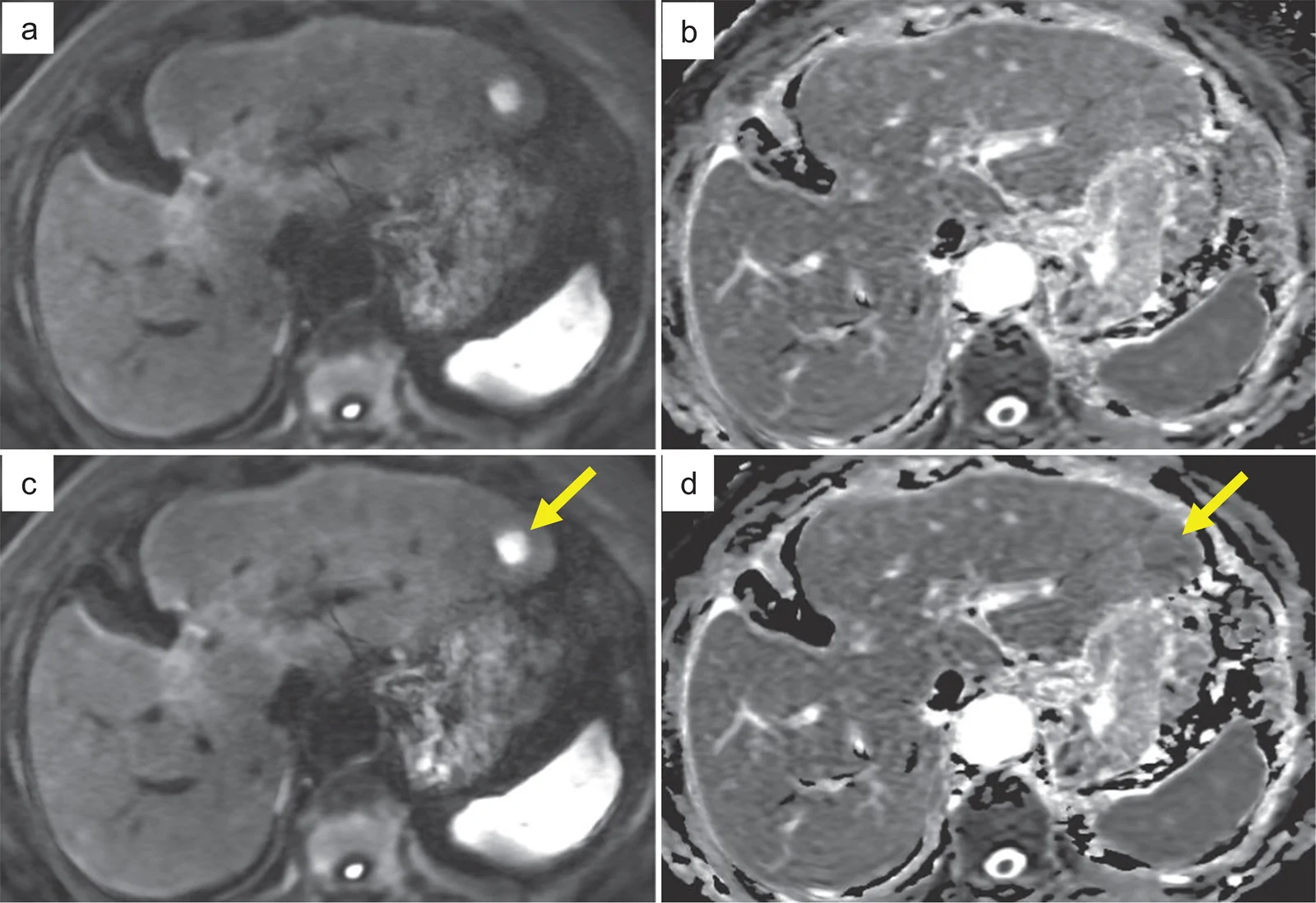
Comparison of (**a**) non-ACSA (conventional) DWI and (**b**) corresponding ADC map and (**c**) ACSA DWI and (**d**) corresponding ADC map (NEX = 4) in a 75-year-old woman with HCC. On ACSA DWI, HCC is clearly seen as a high intensity nodule (arrow in **c**) in the left lateral segment, compared with non-ACSA DWI. On ACSA ADC map, HCC is visualized as a nodule showing mild diffusion restriction (arrow in **d**). ACSA, adaptive complex signal average; ADC, apparent diffusion coefficient; DWI, diffusion-weighted imaging; HCC, hepatocellular carcinoma; NEX, numbers of excitations.

**Fig. 5:**
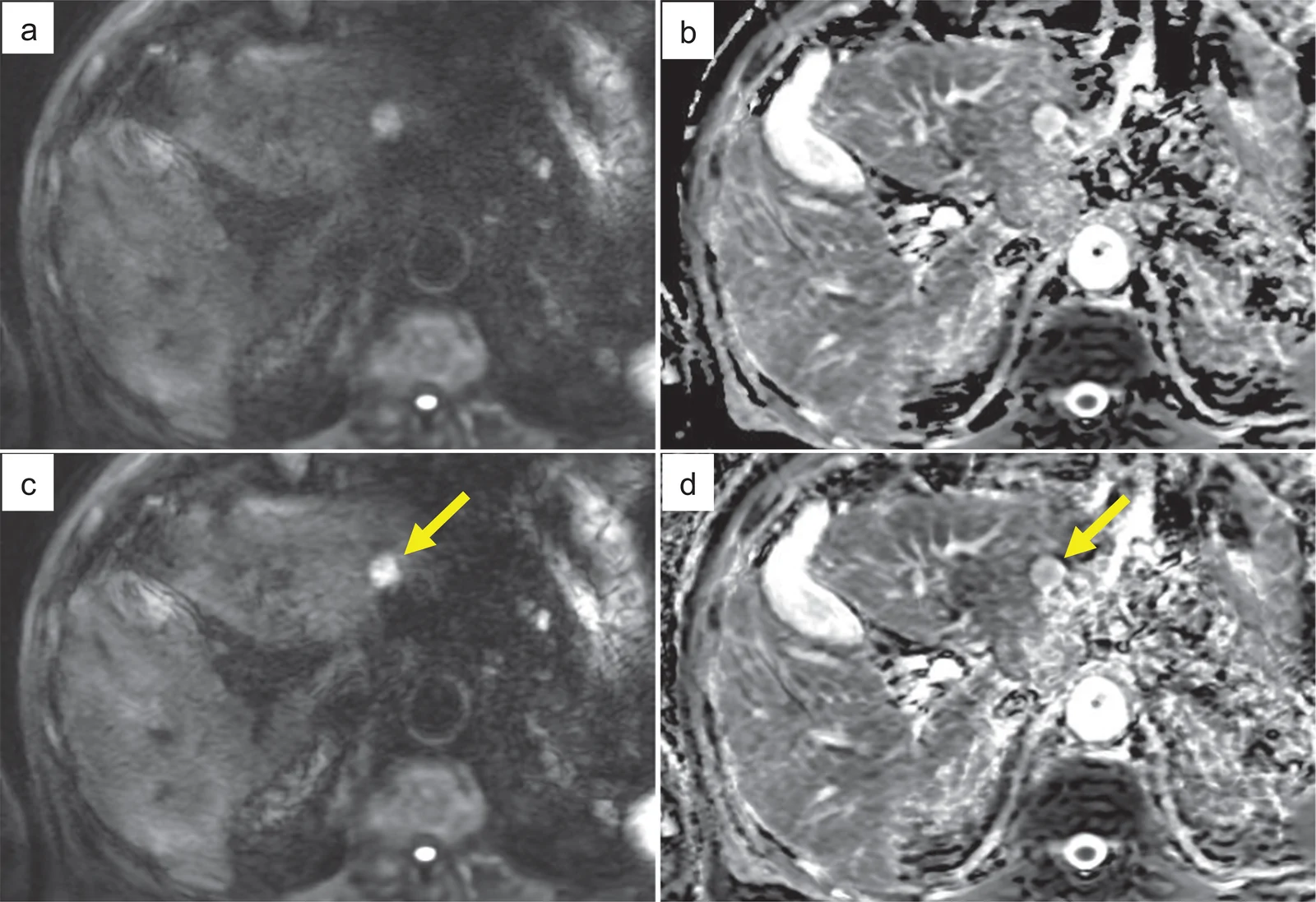
Comparison of (**a**) non-ACSA (conventional) DWI and (**b**) corresponding ADC map and (**c**) ACSA DWI and (**d**) corresponding ADC map (NEX = 3) in a 73-year-old man with hepatic hemangioma. On ACSA DWI, the signal intensity of the liver parenchyma and hepatic hemangioma (arrow in **c**) in the left lateral segment showed a signal increase, compared to non-ACSA DWI. Hemangioma is also depicted as a high signal nodule (arrow in **d**) on the ACSA ADC map. ACSA, adaptive complex signal average; ADC, apparent diffusion coefficient; DWI, diffusion-weighted imaging; NEX, numbers of excitations.

**Table 1:** Comparison of SI of each organ between ACSA DWI and non-ACSA DWI in imaging with NEX 2, 3, or 4

Organs/SI	NEX 2 (n = 21)			NEX 3 (n = 29)			NEX 4 (n = 32)		
	ACSA	Non-ASCA	*P* value	ASCA	Non- ASCA	*P* value	ASCA	Non- ASCA	*P* value
Liver, lateral segment (periphery)	22.9 (14.3)	19.2 (9.7)	< 0.0001	27.1 (17.3)	19.5 (14.1)	< 0.0001	29.1 (18.0)	19.7 (9.9)	< 0.0001
Liver, lateral segment (center)	32.1 (17.3)	28.7 (16.4)	< 0.0001	31.7 (16.3)	27.6 (14.0)	< 0.0001	34.8 (17.3)	27.2 (12.4)	< 0.0001
Liver, right lobe (periphery)	42.7 (27.9)	42.1 (30.2)	0.0020	37.3 (21.6)	37.3 (21.8)	< 0.0001	34.3 (18.2)	34.1 (20.7)	< 0.0001
Liver, right lobe (center)	40.2 (19.5)	40.6 (20.2)	0.5111	38.1 (29.3)	37.2 (28.8)	0.1407	35.0 (23.7)	34.8 (23.2)	< 0.0001
Pancreas, head	65.5 (29.7)	53.1 (30.9)	< 0.0001	58.1 (28.7)	52.3 (26.3)	< 0.0001	58.9 (31.4)	54.9 (30.0)	< 0.0001
Pancreas, body	56.3 (25.9)	56.3 (23.6)	0.0002	61.6 (33.5)	54.9 (29.6)	< 0.0001	61.7 (31.1)	50.1 (25.2)	< 0.0001
Pancreas, tail	60.8 (28.2)	58.8 (25.6)	< 0.0001	68.7 (35.5)	66.4 (29.9)	< 0.0001	58.9 (25.6)	56.8 (22.7)	< 0.0001
Adrenal gland, left	93.4 (17.3)	92.6 (24.9)	0.0103	98.7 (47.0)	89.7 (51.4)	< 0.0001	76.6 (39.8)	68.6 (44.1)	< 0.0001
Spinal erector muscle	29.1 (14.4)	29.1 (14.5)	0.5206	28.6 (13.2)	28.9 (13.4)	0.3300	25.3 (10.2)	25.3 (10.2)	0.7810

Data are medians, with interquartile ranges in parentheses.

ACSA, adaptive complex signal average; DWI, diffusion-weighted imaging; NEX, numbers of excitations; SI, signal intensity.

**Table 2:** Comparison of SNR of each organ between ACSA DWI and non-ACSA DWI in imaging with NEX 2, 3, or 4

Organs/SNR	NEX 2 (n = 21)			NEX 3 (n = 29)			NEX 4 (n = 32)		
	ACSA	Non-ACSA	*P* value	ACSA	Non-ACSA	*P* value	ACSA	Non-ACSA	*P* value
Liver, lateral segment (periphery)	8.5 (5.5)	7.2 (6.0)	0.0001	12.1 (10.5)	8.3 (6.7)	< 0.0001	13.9 (10.7)	9.3 (7.5)	< 0.0001
Liver, lateral segment (center)	12.4 (9.3)	11.6 (10.5)	0.0124	12.5 (14.5)	11.1 (13.1)	< 0.0001	17.8 (14.7)	13.2 (11.7)	< 0.0001
Liver, right lobe (periphery)	17.5 (12.2)	16.6 (14.0)	0.0167	16.9 (15.6)	15.6 (14.5)	0.0003	19.2 (12.0)	18.5 (12.5)	0.3931
Liver, right lobe (center)	17.8 (10.4)	17.4 (12.7)	0.1434	16.4 (15.6)	15.6 (12.9)	0.1033	16.6 (11.2)	17.1 (11.1)	0.4301
Pancreas, head	28.1 (21.1)	27.6 (21.4)	< 0.0001	23.9 (19.4)	21.2 (17.1)	< 0.0001	29.4 (21.1)	29.2 (20.3)	< 0.0001
Pancreas, body	25.5 (20.7)	22.3 (20.6)	0.0009	26.1 (17.7)	22.8 (18.2)	< 0.0001	29.9 (23.2)	26.1 (18.5)	< 0.0001
Pancreas, tail	28.2 (26.6)	27.0 (26.4)	0.0053	27.3 (21.4)	24.9 (24.8)	< 0.0001	30.8 (12.4)	28.1 (17.8)	< 0.0001
Adrenal gland, left	43.0 (20.5)	38.2 (22.9)	0.0466	42.1 (22.9)	37.3 (22.3)	< 0.0001	42.2 (20.1)	41.2 (20.5)	0.0001
Spinal erector muscle	13.5 (5.7)	13.2 (6)	0.2609	12.2 (5.1)	12.4 (4.9)	0.3821	13.7 (5.3)	14.1 (5.3)	0.0007

Data are medians, with interquartile ranges in parentheses.

ACSA, adaptive complex signal average; DWI, diffusion-weighted imaging; NEX, numbers of excitations.

**Table 3:** Comparison of ADC values of each organ between ACSA DWI and non-ACSA DWI in imaging with NEX 2, 3, or 4

Organs/ADC values (10^−6^mm^2^/sec)	NEX 2 (n = 21)			NEX 3 (n = 29)			NEX 4 (n = 32)		
	ACSA	Non-ACSA	*P* value	ACSA	Non-ACSA	*P* value	ACSA	Non-ACSA	*P* value
Liver, lateral segment (periphery)	1375 (505)	1497 (410)	< 0.0001	1176 (417)	1596 (385)	< 0.0001	1185 (340)	1633 (355)	< 0.0001
Liver, lateral segment (center)	1186 (377)	1275 (520)	< 0.0001	1233 (223)	1357 (326)	0.0002	1172 (257)	1345 (353)	< 0.0001
Liver, right lobe (periphery)	1189 (198)	1239 (207)	0.0009	1161 (285)	1175 (309)	< 0.0001	1194 (197)	1204 (223)	< 0.0001
Liver, right lobe (center)	1147 (226)	1170 (220)	0.0046	1122 (275)	1150 (288)	0.0497	1075 (216)	1082 (205)	< 0.0001
Pancreas, head	1193 (234)	1245 (310)	< 0.0001	1237 (305)	1419 (291)	< 0.0001	1125 (222)	1202 (301)	< 0.0001
Pancreas, body	1187 (284)	1364 (335)	0.0002	1244 (287)	1353 (375)	< 0.0001	1285 (340)	1386 (469)	< 0.0001
Pancreas, tail	1137 (178)	1261 (290)	< 0.0001	1263 (355)	1338 (397)	< 0.0001	1140 (170)	1253 (237)	< 0.0001
Adrenal gland, left	1101 (178)	1152 (192)	0.0005	1085 (157)	1132 (195)	< 0.0001	1112 (174)	1178 (163)	< 0.0001
Spinal erector muscle	1568 (117)	1564 (123)	0.5559	1530 (155)	1527 (152)	0.3729	1526 (114)	1528 (115)	0.1067

Data are medians, with interquartile ranges in parentheses.

ACSA, adaptive complex signal average; ADC, apparent diffusion coefficient; DWI, diffusion-weighted imaging; NEX, numbers of excitations.

**Table 4:** Comparison of SIDR of each organ among the differences in NEX 2, 3, or 4

Organs/SIDR	NEX 2(n = 21)	NEX 3(n = 29)	NEX 4(n = 32)	*P* value(Kruskal–Wallis)	*P* value(Steel–Dwass)		
				NEX 2 vs 3 vs 4	NEX 2 vs 3	NEX 2 vs 4	NEX 3 vs 4
Liver, lateral segment (periphery)	11.2 (18.8)	32.4 (33.3)	53.5 (46.8)	< 0.0001	< 0.0001	< 0.0001	0.0428
Liver, lateral segment (center)	3.2 (6.8)	15.0 (14.3)	22.7 (25.2)	< 0.0001	0.0011	< 0.0001	0.4496
Liver, right lobe (periphery)	2.4 (5.2)	2.2 (4.6)	1.1 (4.2)	0.5318			
Liver, right lobe (center)	0.2 (3.0)	0.5 (2.1)	0.8 (3.6)	0.2048			
Pancreas, head	8.6 (14.7)	9.3 (13.4)	7.8 (14)	0.7405			
Pancreas, body	8.7 (16.7)	9.1 (9.4)	9.1 (14.8)	0.6688			
Pancreas, tail	3.4 (17.2)	3.1 (11.7)	11 (10.4)	0.0342	0.9501	0.1143	0.0496
Adrenal gland, left	1.7 (5.3)	6.3 (8.8)	6.0 (9.0)	0.0187	0.0615	0.0190	0.9822
Spinal erector muscle	−0.1 (1.3)	0.0 (0.7)	0.0 (0.7)	0.6268			

Data are medians, with interquartile ranges in parentheses.

NEX, numbers of excitations; SIDR, signal intensity difference ratio.

**Table 5:** Comparison of R–L SI and ADC ratios for ACSA DWI and non-ACSA DWI at NEX of 2, 3, or 4

SI and ADC ratios between right lobe and left lateral segment	NEX 2 (n = 21)			NEX 3 (n = 29)			NEX 4 (n = 32)		
	ACSA	Non-ACSA	*P* value	ACSA	Non-ACSA	*P* value	ACSA	Non-ACSA	*P* value
R–L SI ratio	1.56 (0.97)	1.83 (0.94)	< 0.0001	1.29 (0.49)	1.67 (0.65)	< 0.0001	1.26 (0.50)	1.78 (0.75)	< 0.0001
R–L ADC ratio	0.82 (0.37)	0.75 (0.33)	< 0.0001	0.89 (0.35)	0.77 (0.23)	< 0.0001	0.89 (0.25)	0.64 (0.18)	< 0.0001

Data are medians, with interquartile ranges in parentheses.

ACSA, adaptive complex signal average; ADC, apparent diffusion coefficient; DWI, diffusion-weighted imaging; NEX, numbers of excitations; R–L, right–left; SI, signal intensity.

**Table 6:** Comparison of R–L SI and ADC ratios for ACSA DWI at NEX of 2, 3, or 4

SI and ADC ratios between right lobe and left lateral segment	NEX 2(n = 21)	NEX 3(n = 29)	NEX 4(n = 32)	*P* value(Kruskal–Wallis)	*P* value(Steel–Dwass)		
				NEX 2 vs 3 vs 4	NEX 2 vs 3	NEX 2 vs 4	NEX 3 vs 4
R–L SI ratio	1.56 (0.97)	1.29 (0.49)	1.26 (0.50)	0.0035	0.0108	0.0062	0.9970
R–L ADC ratio	0.82 (0.37)	0.89 (0.35)	0.89 (0.25)	0.0438	0.0542	0.1133	0.7317

Data are medians, with interquartile ranges in parentheses.

ACSA, adaptive complex signal average; ADC, apparent diffusion coefficient; DWI, diffusion-weighted imaging; NEX, numbers of excitations; R–L, right–left; SI, signal intensity.

**Table 7:** Quantitative analysis comparing ACSA DWI and non-ACSA DWI for non-cystic hepatic nodules

Nodule (n = 37)	ACSA	Non-ACSA	*P* value
SI	78.5 (34.5)	73.9 (42.7)	< 0.0001
ADC	1204 (474)	1267 (605)	< 0.0001
CR	2.9 (1.4)	2.8 (1.5)	0.0100
CNR	25.9 (27.3)	22.7 (23.6)	< 0.0001

Data are medians, with interquartile ranges in parentheses.

ACSA, adaptive complex signal average; ADC, apparent diffusion coefficient; CNR, contrast-to-noise ratio; CR, contrast ratio; DWI, diffusion-weighted imaging; SI, signal intensity.
